# Repurposing Oxiconazole against Colorectal Cancer via PRDX2-mediated Autophagy Arrest

**DOI:** 10.7150/ijbs.70679

**Published:** 2022-05-21

**Authors:** Jinyu Shi, Li Zhou, Hui-Si Huang, Liyuan Peng, Na Xie, Edouard Nice, Li Fu, Cen Jiang, Canhua Huang

**Affiliations:** 1 School of Basic Medical Sciences, Chengdu University of Traditional Chinese Medicine, Chengdu, 611137, China.; 2 State Key Laboratory of Biotherapy and Cancer Center, West China Hospital, West China School of Basic Medical Sciences & Forensic Medicine Sichuan University, and Collaborative Innovation Center for Biotherapy, Chengdu, 610041, China.; 3 School of Pharmaceutical Sciences, International Cancer Center, Shenzhen University Health Science Center, Shenzhen 518060, China.; 4 Department of Biochemistry and Molecular Biology, Monash University, Clayton, Victoria 3800, Australia.

**Keywords:** colorectal cancer, oxiconazole, PRDX2, RAB7A, apoptosis, autophagy arrest

## Abstract

Colorectal cancer (CRC) is one of the most common malignancies worldwide, yet successful treatment still remains a challenge. In this study, we found that oxiconazole (OXI), a broad-spectrum antifungal agent, exhibits certain anti-tumor effect against CRC. Autophagy arrest and subsequent apoptosis are characterized as pivotal events involving OXI-induced growth suppression of CRC cells. Mechanistically, OXI downregulates the protein levels of peroxiredoxin-2 (PRDX2), an antioxidant enzyme, for reactive oxygen species (ROS) detoxication, to initiate autophagy by inactivating the Akt/mTOR pathway and inhibiting RAB7A-mediated fusion of autophagosome and lysosome, which lead to extreme accumulation of autophagosomes and subsequent growth suppression of CRC cells. Consistently, interfering with autophagy or overexpressing PRDX2 significantly impedes OXI-induced growth suppression of CRC cells. Moreover, OXI plus oxaliplatin, a mainstay drug for CRC treatment, achieves an improved anti-tumor effect. Taken together, our findings bring novel mechanistic insights into OXI-induced autophagy arrest and the growth inhibitory effect on CRC cells, and suggest a promisingly therapeutic role of OXI for CRC treatment.

## Introduction

Colorectal cancer (CRC) ranks the third in terms of morbidity among all malignant cancers and is the fourth leading cause of death associated with malignancy 1. The incidence is higher in men and increases with age. Although early screening has been shown to reduce CRC incidence and mortality, effective screening programs are still unavailable in many countries 2. Despite surgery, chemotherapy and/or radiation 3 achieve favorable outcome, the first-line chemotherapeutic drugs become compromised because of inevitable side effects and/or drug tolerance. Therefore, the overall survival rate for patients with CRC remains poor 4. Novel therapeutic drugs are therefore required to improve the clinic outcomes for CRC patients.

Autophagy is an evolutionally conserved process which degrades excessive or defective proteins and organelles in autolysosomes after sequestration in autophagosomes, enabling cells to survive stress conditions and maintain energy homeostasis 5. Autophagy exhibits multiple roles in cancer cells due to its context-dependent properties. To date, our group have identified cytoprotective, cytotoxic, nonprotective, and cytostatic autophagy, which are induced by antitumor compounds in various cancer types 6. For example, quercetin elicits protective autophagy in gastric cancer and regorafenib induces cytotoxic autophagy against glioblastoma multiforme, whereas ivermectin exhibits anti-breast cancer activity by activating cytostatic autophagy 7-9. Therefore, elucidating the specific mechanisms underlying the diverse roles played by autophagy in response to drug treatment is scientifically warranted for exploiting autophagy as a therapeutic target in CRC treatment.

Drug repurposing is aimed to search novel applications for drugs beyond the scope of the original purpose for medical use. It has attracted considerable attention in cancer management due to its beneficial therapeutic effects, reduced costs and shorter development timelines 10. Several antibiotic and antifungal agents have demonstrated considerable potential as the candidates for cancer treatment. For example, the antifungal natamycin exhibits antitumor activity and has potential for repurposing as a treatment for HCC; the antibiotic brefeldin A possesses effective antitumor activity in CRC treatment 11, 12. Oxiconazole (OXI), a broad-spectrum antifungal compound derived from imidazole, is currently widely used for the treatment of fungal infections 13. However, the anti-tumor effects of OXI have not yet been investigated.

In this study, we aim to repurpose OXI as a potential candidate for CRC treatment and explore the mechanisms involved, which may provide the molecular basis for applying OXI as a potential therapeutic drug for CRC.

## Materials and Methods

### Cell culture

Human CRC cell lines including HCT116, SW480, RKO, DLD-1, SW620, LoVo and the nonmalignant human colon epithelial cell line NCM460 were cultured in DMEM added with 10% FBS (ExCell Bio), 100 U/ml streptomycin and 100 U/ml penicillin (Beyotime Biotechnology) in a humidified incubator with 5% CO_2_ at 37°C.

### Antibodies and reagents

Reagents were obtained from the following manufacturers:

Oxiconazole nitrate (OXI; HY-B1324), Chloroquine (CQ; HY-17589), 3-methyladenine (3-MA; HY19312), Rapamycin (RAPA; HY-10219) and oxaliplatin (HY-17371) were purchased from Med Chem Express. Z-VAD-FMK (S7023) and N-acetyl cysteine (NAC; A7250) were purchased from Selleck. Dimethyl sulfoxide (DMSO; D2650), Crystal Violet (C0775) and 3-(4,5-dimethylthiazol-2-yl)-2,5-diphenyltetrazolium bromide (MTT; M2128) were obtained from Millipore Sigma. DAPI (62248) and Lipofectamine 3000 (L3000015) were obtained from Thermo Fisher Scientific. RAPA, Z-VAD-FMK and OXI were dissolved in DMSO. CQ, NAC, oxaliplatin, 3-MA, Crystal Violet and MTT were dissolved in Phosphate Buffer Saline (PBS).

The antibodies were as follows:

LC3B (NB100-233) was obtained from Novus Biologicals (Saint Charles, MO, USA); anti-ATG5 (12994S), anti-Beclin1 (3738), anti-Bcl-2 (15071), anti-p-Akt (4060S), anti-Akt (4685S), anti-mTOR (2972S), anti-p-mTOR (2971S), anti-p70S6K (9202S), anti-p-p70S6K (9208S), anti-p-4EBP1 (9451S), anti-caspase 3 (9662S), anti-4EBP1 (9452S) and anti-cleaved-caspase 3 (9664S) were purchased from Cell Signaling Technology; anti-PRDX2 (ab109367) and anti-Ki67 (ab16667) were obtained from Abcam; anti-β-actin (sc-1616), anti-p62 (sc-48402), anti-SNAP29 (sc-135564), anti-Myosin (sc-393053), anti-Dynactin (sc-365274), anti-Dynein HC (sc-514579) and anti-RAB7A (sc-376362) were purchased from Santa Cruz Biotechnology.

### Detection of cell growth and proliferation

The MTT assay was conducted to evaluate the cell growth rate. Cells were seeded in a 96-well plate (4000 cells/well) and incubated for 24 h with indicated concentrations of drug. The absorbance of each well was detected by a spectrophotometry at 570 nm wavelength. As for colony formation assay, CRC cells were seeded in a 24-well plate (500 cells/well) for individual treatments. After treatment of one week, 4% paraformaldehyde (PFA) was used to fix cells and crystal violet was used to dye the cells. After washing three times with water, dyed cells were dissolved in 0.1% Sodium dodecyl sulfate (SDS). Then, the absorbance was measured at 570 nm reference wavelength with microplate reader. The 5-Ethynyl-2′-deoxyuridine (EdU) incorporation assay kit (Ribobio) was used to detect cell proliferation as described previously 8.

### Lactate dehydrogenase (LDH) release assay

LDH release assay was used to assess the cytotoxicity of OXI. CRC cells were cultured in a 96-well plate (5×10^3^ cells/well). Then, CRC cells were incubated with indicated concentrations of OXI for 24 h after cells grew to about 80% confluency. The supernatant of cultured cells was transferred to a new 96-well plate for detection using a LDH test kit (Beyotime Biotechnology).

### Detection of cellular ROS levels

The ROS levels were monitored using an active oxygen analysis kit (Beyotime Biotechnology) as recommended by the manufacturers. CRC cells were incubated in a 6-well plate (3×10^5^ cells/well), and then treated with different concentrations of OXI for 24 h. After that, cells were dyed with Muse™ Oxidative Stress reagent. Then, dyed cells were collected for intracellular ROS detection using a FACS Calibur flow cytometer (BD Biosciences) (see below).

### Flow Cytometry

The induction of apoptosis was detected using an annexin V/ PI Detection Kit (KeyGen Biotech) as recommended by the manufacturers. The treated CRC cells were collected and washed with PBS. Then the cells were resuspended in binding buffer. Following incubating the cell suspension with annexin V-FITC and PI-PE, 2×10^4^ living cells were collected on a FACS Calibur flow cytometer (BD Biosciences). All data from flow cytometry were analyzed using FlowJo software.

### Western blotting analysis

Cells were collected and lysed with RIPA buffer supplemented with phosphatase inhibitor and protease inhibitor for 30 min on ice. The lysates were quantified using the Bradford Protein Assay Kit (Bio-Rad). The lysed samples were then undergone SDS-PAGE and incubated with the different primary and secondary antibodies. The relative protein intensity was visualized using a ChemiScope 6000 Touch chemiluminescence imaging system (Clinx).

### Label-free quantitative proteomics

HCT116 cells were harvested and lysed after treatment with DMSO or 30 μM OXI. Then, the protein lysates were reduced and alkylated with Tris (2-carboxyethyl) phosphine (TCEP) and Iodoacetamide (IAA). After digestion with trypsin, samples were pooled and dried. Peptides were desalted using C18 Stage Tips, suspended in 2% acetonitrile (ACN) and 0.1% trifluoroacetic acid (TFA), and finally analyzed by LC-MS using a Thermo Fisher Scientific Q Exactive Plus.

### Immunofluorescence

CRC cells were grown on sterilized cover slips in a 24-well plate (5×10^3^ cells/well), fixed with 4% PFA after indicated treatment. After washing twice with PBS, cells were blocked and permeabilized with goat serum and Triton X-100, respectively. Indicated primary antibodies were incubated within the cells overnight, followed by incubation of Alexa Fluor secondary antibody for 1-2 h. Then, nuclei were stained with DAPI. Images were obtained by confocal microscopy (Carl Zeiss).

### RNA interference

The siRNAs of *ATG5*, *BECN1* and *PRDX2* were purchased from Gene Pharma. The sequences were as follows: human *ATG5*, 5'-GCAACUCUGGAUGGGAUUGTT -3'; human* BECN1*, 5'-CAGUUUGGCACAAUCAAUATT-3'; human *PRDX2*, 5'-GCAACGCGCAAAUCGGAAATT-3'.

The siRNAs were transfected using Lipofectamine 3000 reagent, and the detailed operations were performed as the manufacturer's protocol.

### Generation of tumor xenograft model

The BALB/c nude mice (six-week-old) were purchased from HFK Bioscience and raised under SPF conditions. For the generation of subcutaneous xenograft model, HCT116 cells (1×10^7^/mouse) were injected subcutaneously into mice. After the tumor volume reached about 100 mm^3^, mice were divided into two groups and intraperitoneally injected with OXI (50 mg/kg/d) or vehicle (5% DMSO, 10% ethanol, 10% ricinus oil and 75% physiologic saline). The tumor volumes were detected every day and calculated by using the following formula: (L × W^2^ /2). Mice were euthanized after 12 days and xenograft tumors were harvested. All animal studies in the present work were approved by the related committee of Sichuan University.

### Immunohistochemistry (IHC)

IHC staining was performed as previously described 14. The staining intensity was divided into four grades (A: 0, negative; 1, weakly positive; 2, positive; 3, strongly positive) and the percentage of staining-positive cells was indicated by five grades (B: 0, <5%; 1, 6-25%; 2, 26-50%; 3, 51-75%; 4, >75%). The final expression scores were calculated through multiplying A by B.

### Statistical analysis

Statistical analysis was performed by using GraphPad Prism Software. One-way ANOVA or two-tailed Student's t test was used to analyze the statistical differences. All data were presented as means ± SD and *P* < 0.05 was defined as statistically significant.

## Results

### OXI suppresses the growth of CRC cells both *in vitro* and *in vivo*

**Figure 1:**
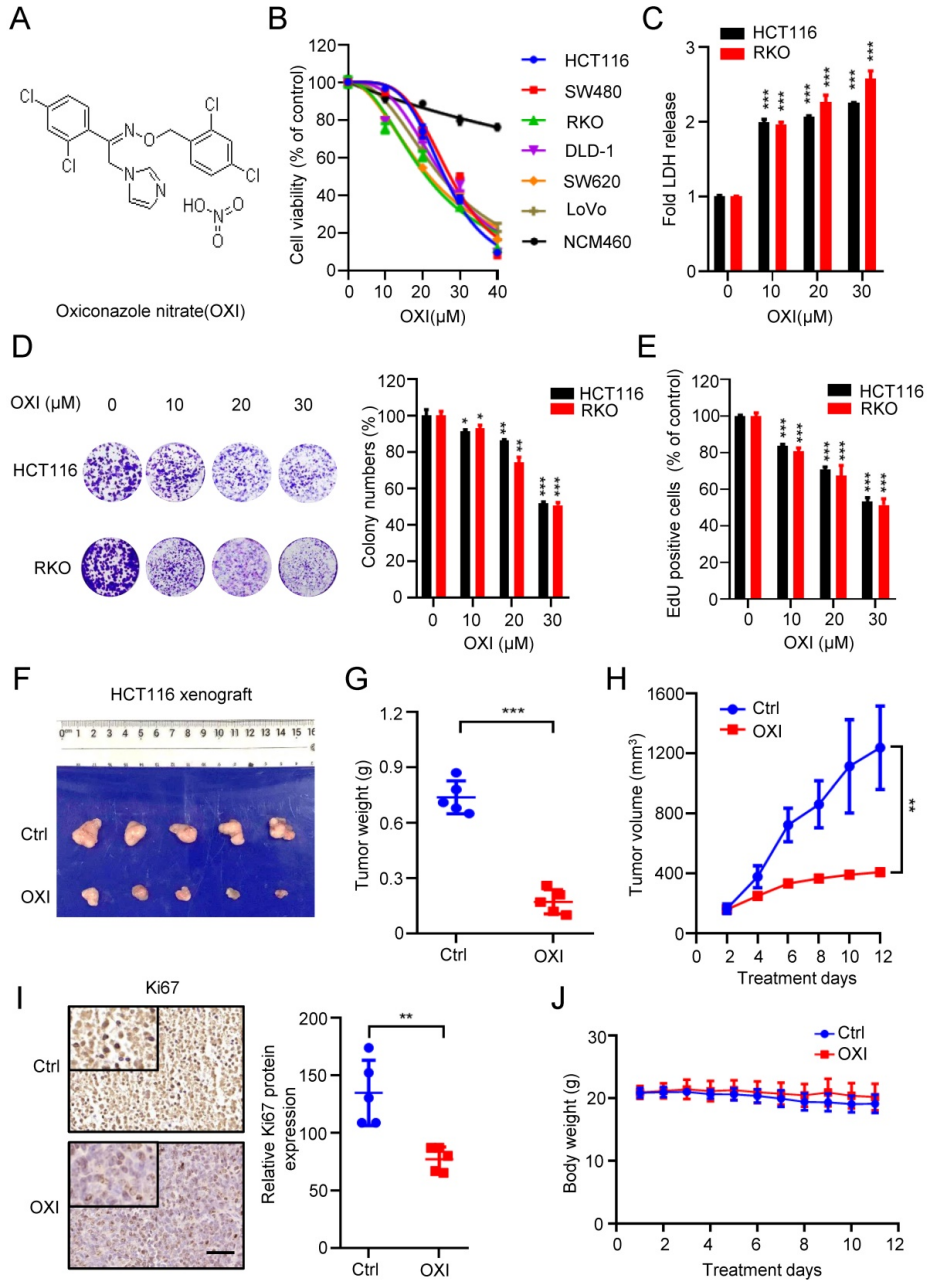
** Oxiconazole inhibits CRC cell growth both *in vitro* and *in vivo*.** (A) Chemical structure of OXI. (B) MTT assay of CRC cells after treatment with different concentrations of OXI for 24 h. IC50 (μM): HCT116, 25.86; SW480, 27.34; RKO, 21.01; DLD-1, 25.56; SW620, 21.75; LoVo, 24.87; NCM460, 126.4. (C) HCT116 and RKO cells were incubated with OXI, LDH release analysis was used to assess the cytotoxicity of OXI. (D, E) RKO and HCT116 cells were subjected to different concentrations of OXI. Colony formation assay (D) and EdU incorporation assay (E) were performed to detect the cell proliferation. (F) Representative images of tumors (N=5) from vehicle or OXI-treated (50 mg/kg/day) mice were shown. (G) The tumor weight in F is shown. (H) Tumor volumes of vehicle- or OXI-treated mice. (I) The Ki67 expression in tumor xenografts. Scale bar: 50 μm. (J) Body weights of mice in F measured at the indicated time. All data are means ± SD.

To validate whether OXI (Fig. 1A) displays an anticancer activity against CRC, we first detected cell growth of OXI-treated CRC cells. As depicted in Fig. 1B, 24 h-treatment of OXI significantly inhibited the growth of several kinds of CRC cells, including HCT116, RKO, DLD-1, SW620, SW480 and LoVo. On the contrary, OXI showed little cytotoxicity in NCM460 cells (the nonmalignant human colon epithelial cell line). Consistently, results from LDH release assay found that treatment with OXI displayed significant cytotoxicity in CRC cells (Fig. 1C). In addition, the colony formation assay (Fig. 1D) and EdU incorporation assay (Fig. 1E) indicated the proliferation-suppressive effect of OXI on CRC cells. In summary, these data suggest that OXI shows anticancer effect in CRC cells *in vitro*.

To confirm the antitumor activity of OXI on CRC *in vivo*, we generated a CRC xenograft model by inoculating HCT116 cells subcutaneously into nude mice. We found that OXI treatment reduced the tumor size, tumor weight and growth rate of CRC xenografts (Fig. 1F-H). Consistently, immunohistochemistry staining showed weaker Ki67 intensity in OXI treated group than those in control group (Fig. 1I). These findings indicate that OXI treatment significantly restrains CRC cell growth *in vivo*. Furthermore, we found that OXI exposure display no obvious changes on the weight of mice (Fig. 1J), or pathological alterations of major organs (Fig. S1), indicating that OXI shows no obvious side effects on mice. Together, these findings suggest that OXI suppresses the growth and proliferation of CRC cells both *in vitro* and *in vivo*.

### OXI inhibits CRC cell growth by triggering apoptosis

**Figure 2:**
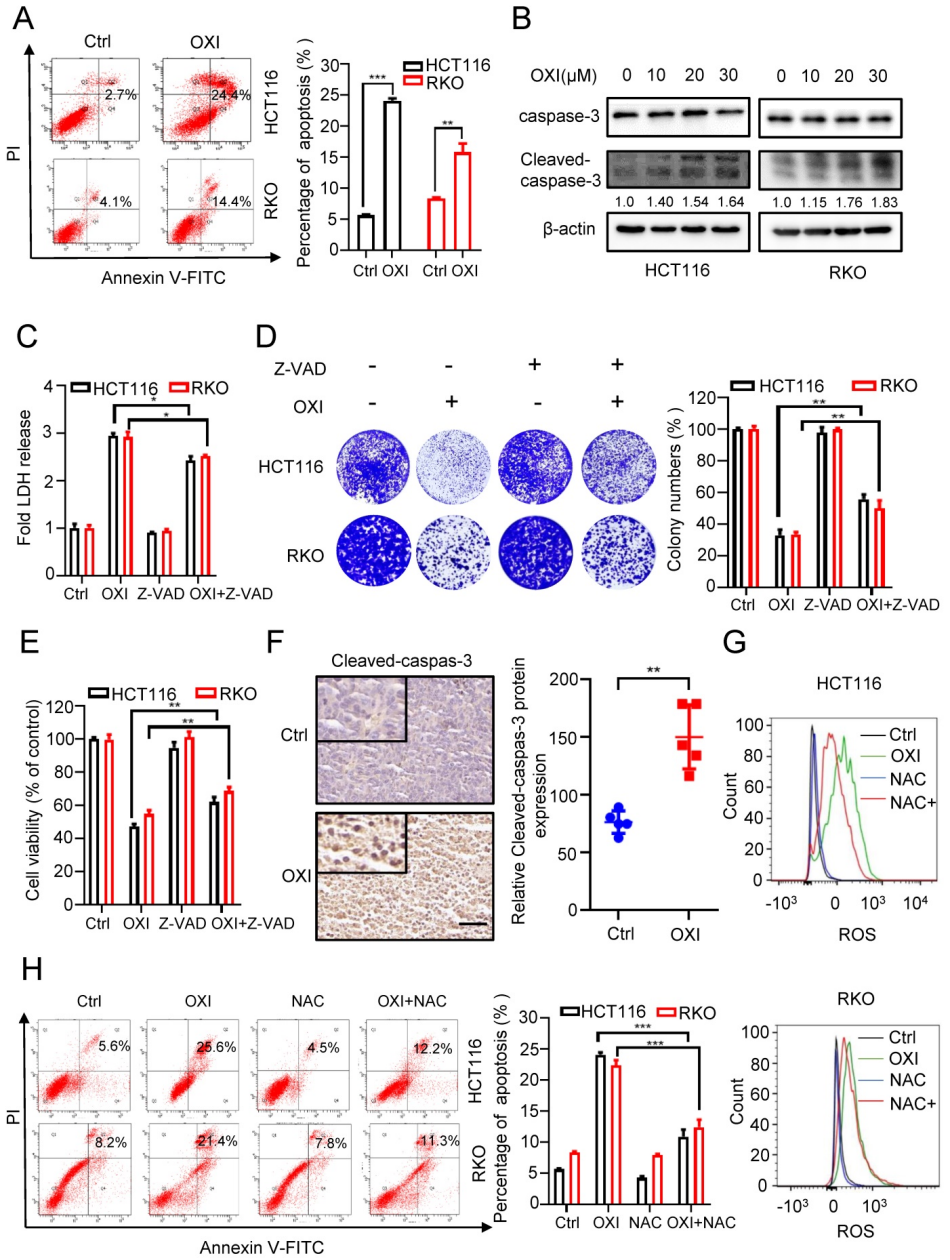
** OXI inhibits cell growth by promoting apoptosis of CRC cells.** (A) Annexin V/PI staining and (B) Cleaved-caspase-3 levels were used to detect apoptosis induced by OXI in CRC cells. (C) LDH release assay, (D) Colony formation assay and (E) MTT assay of HCT116 and RKO cells after treatment with OXI in the presence or absence of Z-VAD for 24 h. (F) Relative cleaved-caspase-3 protein expression in xenograft tumors was evaluated by IHC staining. Scale bar: 50 μm. All data are means ± SD. (G) Representative cellular ROS levels were determined by flow-cytometric analysis in RKO and HCT116 cells treated with OXI (30 μM) in combination with or without NAC (2.5 mM). (H) Annexin V/PI staining of apoptosis in HCT116 and RKO cells treated as in G by flow cytometry.

To unearth the underlying mechanism of OXI-induced growth suppression, we investigated whether OXI treatment influences CRC cell apoptosis. Firstly, we measured apoptotic cells using Annexin V/PI staining. As show in Fig. 2A, the results showed a significant increase of apoptotic CRC cells after OXI treatment. Consistently, the cleavage of caspase 3, a marker of cell apoptosis, was also increased in OXI-treated CRC cells (Fig. 2B). To further confirm OXI-induced apoptosis, HCT116 and RKO cells were treated with the apoptosis inhibitor Z-VAD-FMK plus OXI. The pro-apoptotic effect of OXI was partially inhibited by Z-VAD-FMK, as evidenced by LDH release, colony formation and MTT assays (Fig. 2C-E). To validate the pro-apoptotic activity of OXI *in vivo*, the intensity of cleaved-caspase 3 in tissues from mice xenografts was detected. As expected, OXI-treated xenografts exhibited stronger cleaved-caspase 3 intensity than vehicle-treated group (Fig. 2F). In brief, these results reveal that apoptosis may be involved in OXI-induced growth suppression of CRC cells*.* As excessive ROS production may be a possible reason for apoptosis induction 15, we monitored whether OXI treatment increases ROS production. As shown in Fig. 2G, an increased level of ROS was detected in OXI-treated CRC cells. Consistently, combinational treatment of OXI with ROS scavenger (NAC, N-acetylcysteine) marked reversed OXI-induced ROS accumulation in CRC cells (Fig. 2G). Furthermore, treatment with NAC also decreased the pro-apoptotic effect of OXI (Fig. 2H). Together, these findings suggest that OXI enhances apoptosis in CRC cell by promoting cellular ROS production.

### OXI initiates autophagy and inhibits fusion of autophagosome and lysosome in CRC cells

**Figure 3:**
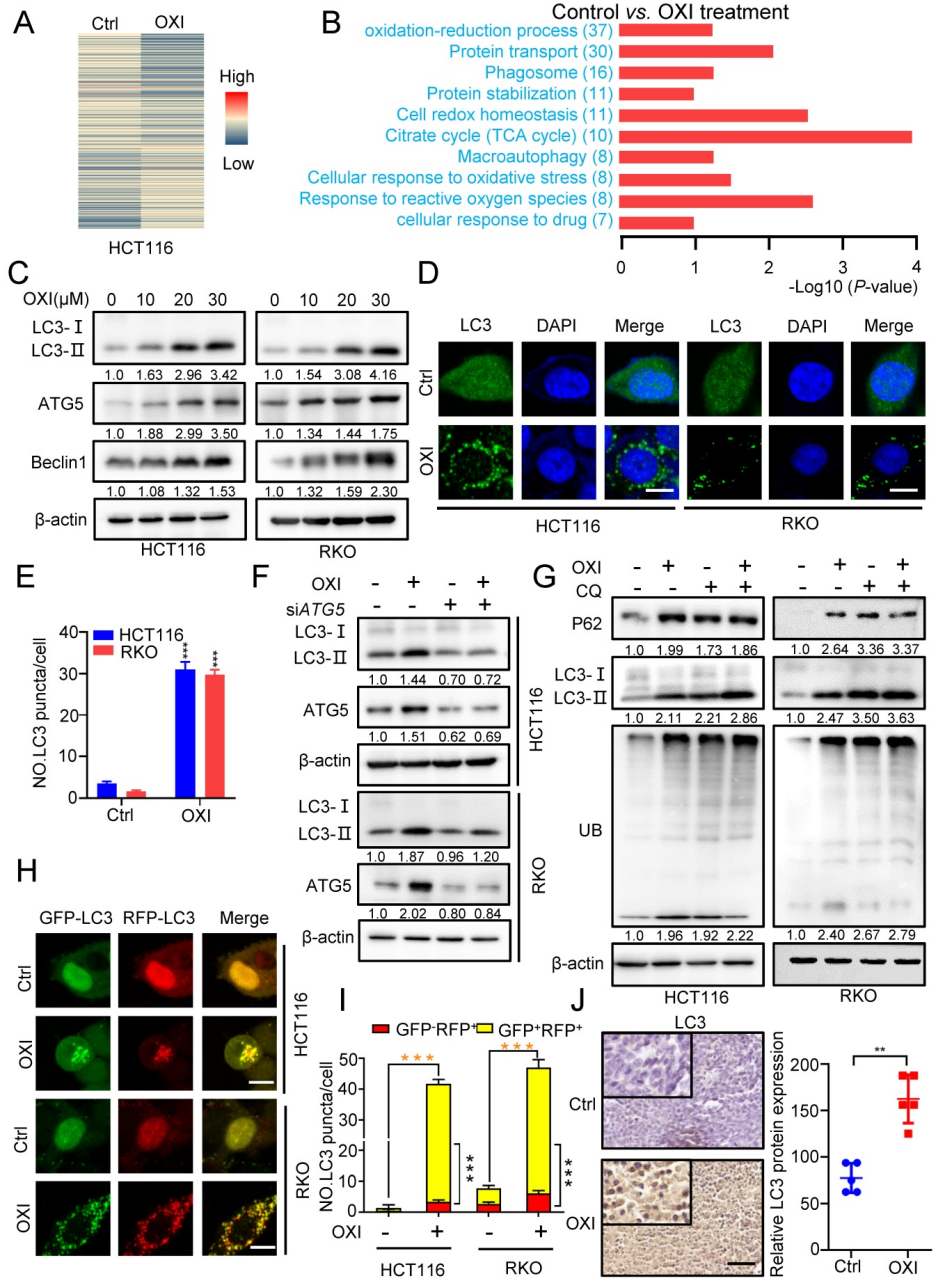
** OXI initiates autophagy and inhibits fusion of autophagosome and lysosome in CRC cells.** (A) Normalized intensity values of upregulated and downregulated proteins in HCT116 cells treated with OXI (30 μM) were shown in heat map. (B) KEGG pathway and GO analyses of changed proteins in A. (C) Western blotting analysis of LC3 turnover, ATG5 and Beclin1 protein levels in OXI-treated CRC cells. (D) LC3 puncta in RKO and HCT116 cells were detected by immunofluorescent analysis after treatment of DMSO or OXI (30 μM). (E) Quantification of LC3 puncta. Scale bars, 10 μm. (F) Western blotting analysis of LC3 turnover in HCT116 and RKO cells transfected with si*ATG5* or si*Scramble* and treated with OXI (30 μM). (G) Western blotting analysis of LC3 turnover, p62 and ubiquitinated proteins in HCT116 and RKO cells treated with OXI (30 μM) in combination with CQ (10 μM) for 24 h. (H) IF analysis of HCT116 and RKO cells transfected with the tandem mRFP-GFP-tagged LC3B plasmids and treated with OXI (30 μM). Scale bars, 10 μm. (I) Quantification of the ratio of yellow puncta (autophagosome) versus red puncta (autolysosome). (J) The expression of LC3 in xenografts was evaluated by IHC. Scale bar: 50 μm. All data are means ± SD.

To further elucidate the underlying mechanism of OXI-induced growth inhibition, we performed the label-free quantitative proteomics to monitor the overall changes of protein expression in OXI treated HCT116 cells (Fig. 3A). KEGG pathway and Gene ontology (GO) analysis identified the differential expressed proteins, and the oxidation-reduction process, protein transport and phagosome formation were enriched (Fig. 3B).

We therefore wondered whether the autophagy process is involved in the anti-tumor effect of OXI in CRC cells. Firstly, we detected the expression of autophagy-related genes after OXI treatment. As expected, the LC3 turnover (LC3-I to lipidated LC3-II) was enhanced by OXI treatment in HCT116 and RKO cells (Fig. 3C and Fig. S2A). We also detected the protein levels of ATG5 and Beclin1 to determine whether OXI promotes the autophagic vesicles formation. As depicted in Fig. 3C, the protein levels of ATG5 and Beclin1 were enhanced upon OXI treatment in a dose-dependent manner. Moreover, the autophagy was further confirmed by the increase of LC3B puncta in OXI-treated CRC cells (Fig. 3D-E). Moreover, interfering the expression of either *ATG5* or *Beclin1* with siRNA in CRC cells significantly restored the turnover of LC3 and accumulation of LC3 puncta after OXI treatment (Fig. 3F and Fig. S2B-E). Previous studies have confirmed that impaired interaction of Belin1 with Bcl-2 is pivotal during autophagy initiation 11. We found that the interaction of Belin1 with Bcl-2 was significantly decreased by OXI treatment in CRC cells (Fig. S2F). In summary, these data indicate that OXI treatment initiates autophagy in CRC cells.

To reveal whether OXI promotes the late stage of autophagy, we detected the expression levels of LC3B, p62 (known as a substrate of autophagy) and ubiquitinated proteins by combinational use of CQ in OXI-treated cells. We observed LC3B accumulation along with increased p62 levels in OXI-treated cells, as well as an increase of ubiquitinated proteins (Fig. 3G), suggesting the blockage of autophagic flux. In addition, we used tandem monomeric mRFP-GFP tagged LC3 plasmid (in which the GFP fluorescence of mRFP-GFP-LC3B tandem was quenched whereas RFP fluorescence was stable in acidic compartments) to detect the formation of autolysosomes in OXI-treated CRC cells. OXI treatment increased the GFP^+^RFP^+^ signal (autophagosome) rather than GFP^-^RFP^+^ signal (autolysosome) in CRC cells, which implied increased autophagosomes and impaired autophagic flux in CRC cells (Fig. 3H-I). Consistently, OXI-treated xenografts exhibited stronger LC3 intensity (Fig. 3J). Together, our data reveal that OXI impairs the fusion of autophagosome and lysosome in CRC cells, leading to restrained autophagic flux.

### Autophagy induction is essential for the antitumor activity of OXI

**Figure 4:**
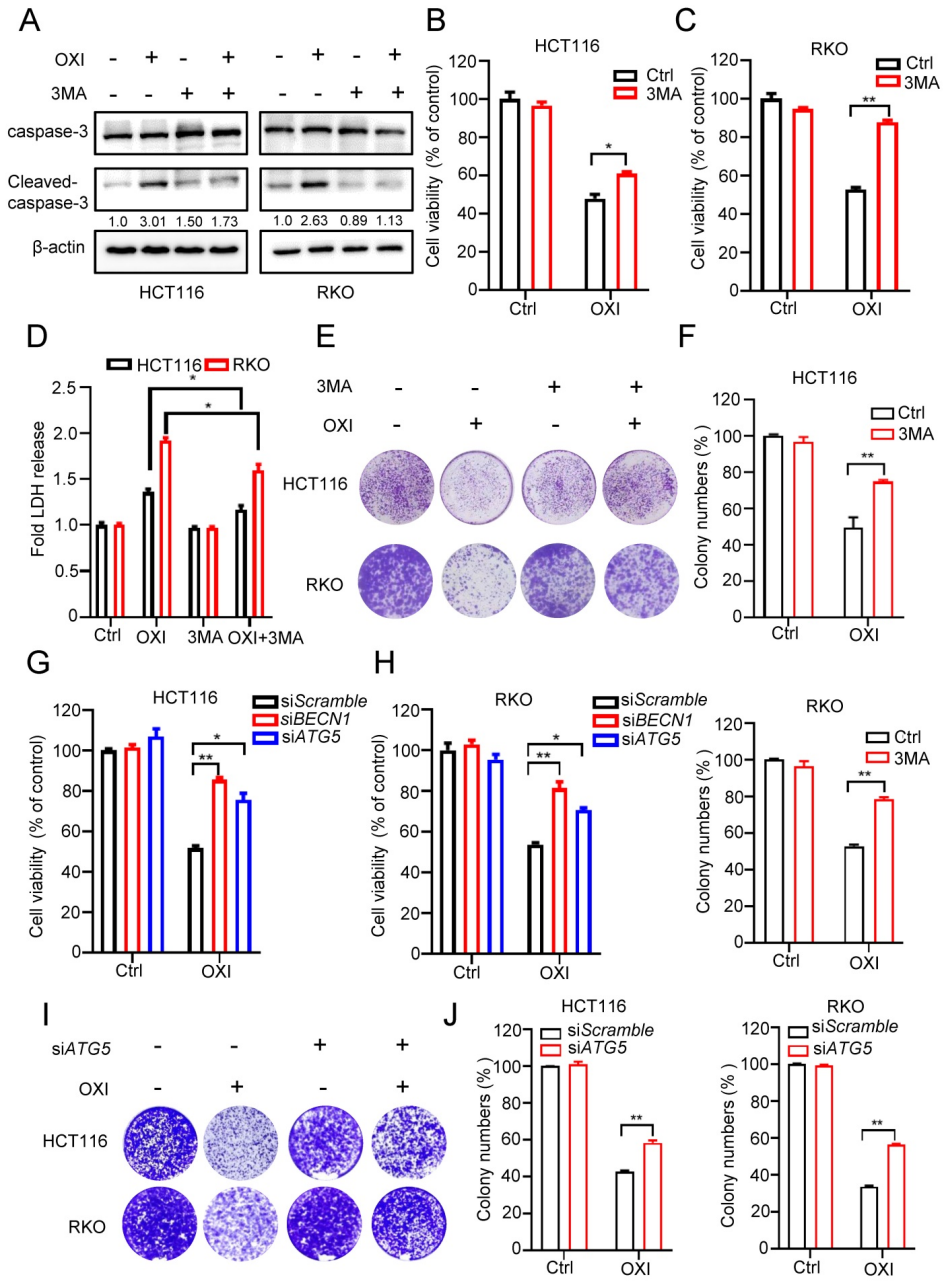
** Autophagy induction is essential for the antitumor activity of OXI in CRC cells.** (A) Western blotting analysis of Cleaved-caspase-3 levels in HCT116 and RKO cells after combinational treatment of OXI (30 μM) and 3MA (1 mM) (B-C) Cell growth of HCT116 and RKO cells after treatment with OXI (30 μM) in the presence of 3-MA (1 mM) for 24 h. (D) LDH release assay in HCT116 and RKO cells treated as in B and C. (E-F) Colony formation assay was performed to detect cell proliferation. HCT116 and RKO cells were treated with OXI (30 μM) in the absence or presence of 3-MA (1 mM) for 2 weeks. (G-H) MTT assay of HCT116 and RKO cells transfected with si*BECN1* or si*ATG5* and treated with OXI (30 μM). (I-J) Colony formation assay of HCT116 and RKO cells transfected with si*Scramble* or si*ATG5* followed by OXI (30 μM) treatment for 2 weeks. All data are means ± SD.

To demonstrate the effect of autophagy induction on the anti-CRC activity of OXI, HCT116 and RKO cells were treated with OXI in combination with or without 3-MA (an inhibitor of autophagy induction). Firstly, we detected apoptosis in CRC cells after combinational treatment of OXI and 3MA, western blot analysis revealed that combinational use of 3-MA with OXI restored OXI-mediated apoptosis (Fig. 4A). As depicted in Fig. 4B and C, combinational use of 3-MA with OXI partly restored OXI-mediated CRC growth inhibition. Moreover, LDH release assay and colony formation analysis also revealed that 3-MA crippled OXI-induced cytotoxicity and proliferative suppression (Fig. 4D-F). In addition to pharmacological inhibition, we also used siRNAs to silence autophagy genes (*Atg5* and *Beclin1*) for further verifying the role of OXI-induced autophagy. As shown in Fig. 4G and H, siRNA-targeted *Atg5* and *Beclin1* knockdown counteracted OXI-induced growth suppression of CRC cells. Similar observations on cell proliferation were also observed using this treatment, as demonstrated by the results from the colony formation assay (Fig. 4I and J). In conclusion, these findings indicate that OXI-regulated autophagy induction is involved in CRC suppression.

### OXI promotes the initiation of autophagy by inhibiting Akt/mTOR axis

As the Akt/mTOR axis, a canonical negative regulatory pathway for autophagy induction, is also correlated with tumor proliferation 16, we questioned whether Akt/mTOR axis is required for OXI-regulated autophagy initiation. As depicted in Fig. S3A, OXI treatment suppressed the activation of Akt/mTOR axis in CRC cells, as demonstrated by reduced phosphorylation level of Akt and mTOR, as well as the downstream effector 4E-BP1 and p70S6K. Moreover, we transfected the CA-Akt plasmids (a constitutively active form of Akt) into CRC cells to explore the role of the Akt/mTOR axis in OXI-regulated autophagy. As expected, reactivation of Akt enforced an increase of phosphorylated Akt and markedly reversed OXI-induced LC3 turnover and LC3B puncta in OXI treated CRC cells (Fig. S3B-D). These results together suggest the essential role of Akt/mTOR axis in OXI-induced autophagy initiation in CRC cells.

### Downregulation of PRDX2 is involved in OXI-induced autophagy initiation and anticancer effects

**Figure 5:**
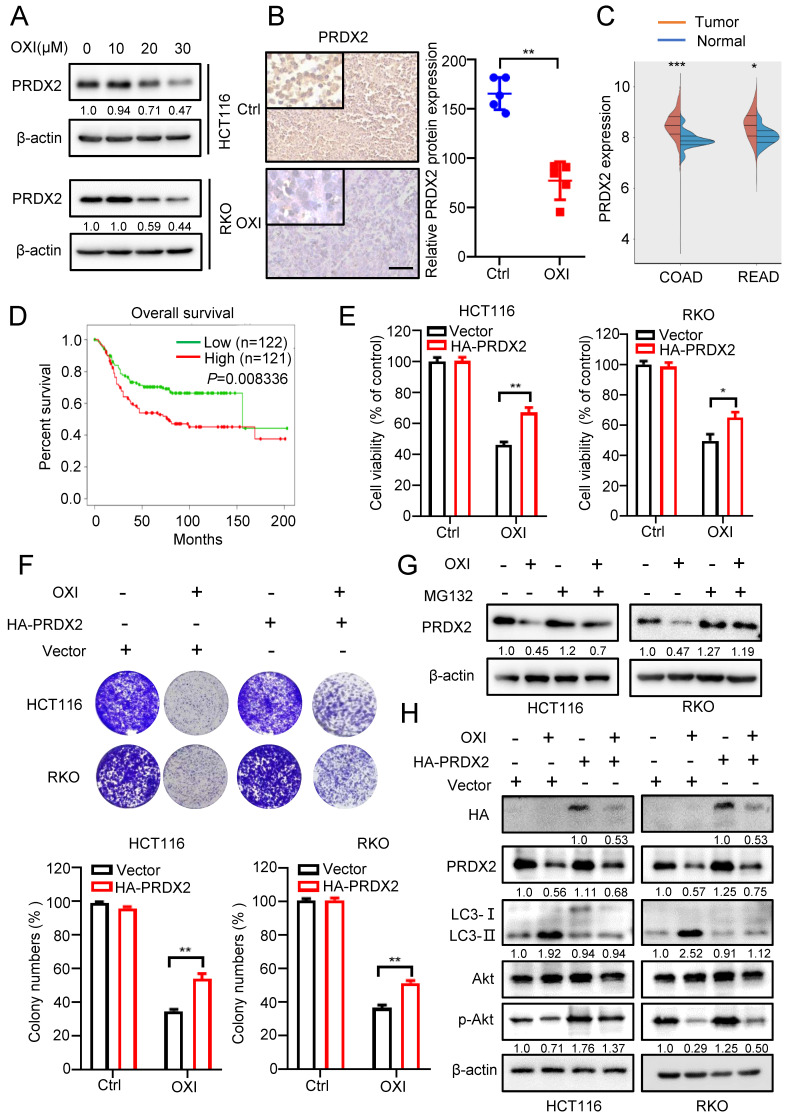
** Downregulation of PRDX2 is involved in OXI-induced autophagy initiation and anti-CRC effects.** (A) The protein levels of PRDX2 in RKO and HCT116 cells treated with different concentrations of OXI. (B) The protein levels of PRDX2 in xenografts were evaluated by IHC staining. Scale bar: 50 μm. (C-D) PRDX2 expression in tumor/normal and KM analysis of patients' survival based on PRDX2 expression in TCGA (colorectal cancer) dataset. (E) MTT assay of HCT116 and RKO cells transiently transfected with HA-PRDX2 and treated with OXI (30 μM). (F) Colony formation assay of HCT116 and RKO cells treated as in E. (G) HCT116 and RKO cells were treated with OXI (30 μM) for 24 h, followed by treatment with MG132 (10 μM) for 4 h. PRDX2 protein levels were measured by western blotting. (H) Western blotting analysis of the protein levels of PRDX2, Akt, p-Akt and LC3 in RKO and HCT116 cells transfected with Vector or HA-PRDX2 and treated with OXI (30 μM). All data are means ± SD.

Previous reports have indicated that downregulation of PRDXs is associated with impaired antioxidant response and oxidative stress 17. According to our previous proteomics data, we found that the PRDX2 proteins were markedly downregulated following the treatment with OXI. Consistently, as depicted in Fig. 5A, OXI treatment markedly decreased PRDX2 protein level in RKO and HCT116 cells. In line with this, tissues from OXI-treated xenografts displayed weaker PRDX2 staining (Fig. 5B). Importantly, the protein level of PRDX2 in normal human tissues was lower than that in CRC patients (Fig. 5C). Furthermore, data from TCGA indicated that higher PRDX2 expression in CRC patients was correlated with poorer disease-free survival (*P* = 0.008) (Fig. 5D). Therefore, we determined whether OXI-induced CRC suppression is attributable to PRDX2 downregulation caused by OXI treatment. HA-tagged PRDX2 plasmid was transfected into RKO and HCT116 cells to detect CRC cell growth under OXI treatment. As shown in Fig. 5E and F, PRDX2 overexpression reversed OXI-induced suppression of cell proliferation, as evidenced by results from the colony formation assay and MTT assay. We next addressed whether OXI-induced downregulation of PRDX2 was caused by proteasome/ubiquitination-mediated degradation. The results demonstrated that MG132 (a proteasome inhibitor) treatment restored OXI-inhibited protein levels of PRDX2, indicating that PRDX2 might be degraded by the proteasome (Fig. 5G).

In addition, mounting evidence has found that the protein levels of PRDX2 are positively correlated with the activation of Akt 18-20. We therefore questioned whether OXI-induced downregulation of PRDX2 contributes to the inactivation of Akt/mTOR axis and subsequent autophagy initiation. As shown in Fig. 5H and Fig. S4A, overexpression of PRDX2 partly reversed OXI-mediated inhibition of Akt phosphorylation and thus inhibited the LC3 turnover and LC3 puncta. In contrast, silencing PRDX2 promoted the turnover of LC3 and the formation of LC3B puncta (Fig. S4B-S4C). These data suggest that OXI downregulates PRDX2 to promote autophagy initiation and suppress CRC cell growth.

### OXI inhibits autophagosome-lysosome fusion by repressing PRDX2/RAB7A axis in CRC cells

To gain more mechanistic insights into OXI-regulated inhibition of lysosome-autophagosome fusion, we further detected the lysosomal mass by Lyso-Tracker Red staining in OXI-treated CRC cells.

**Figure 6:**
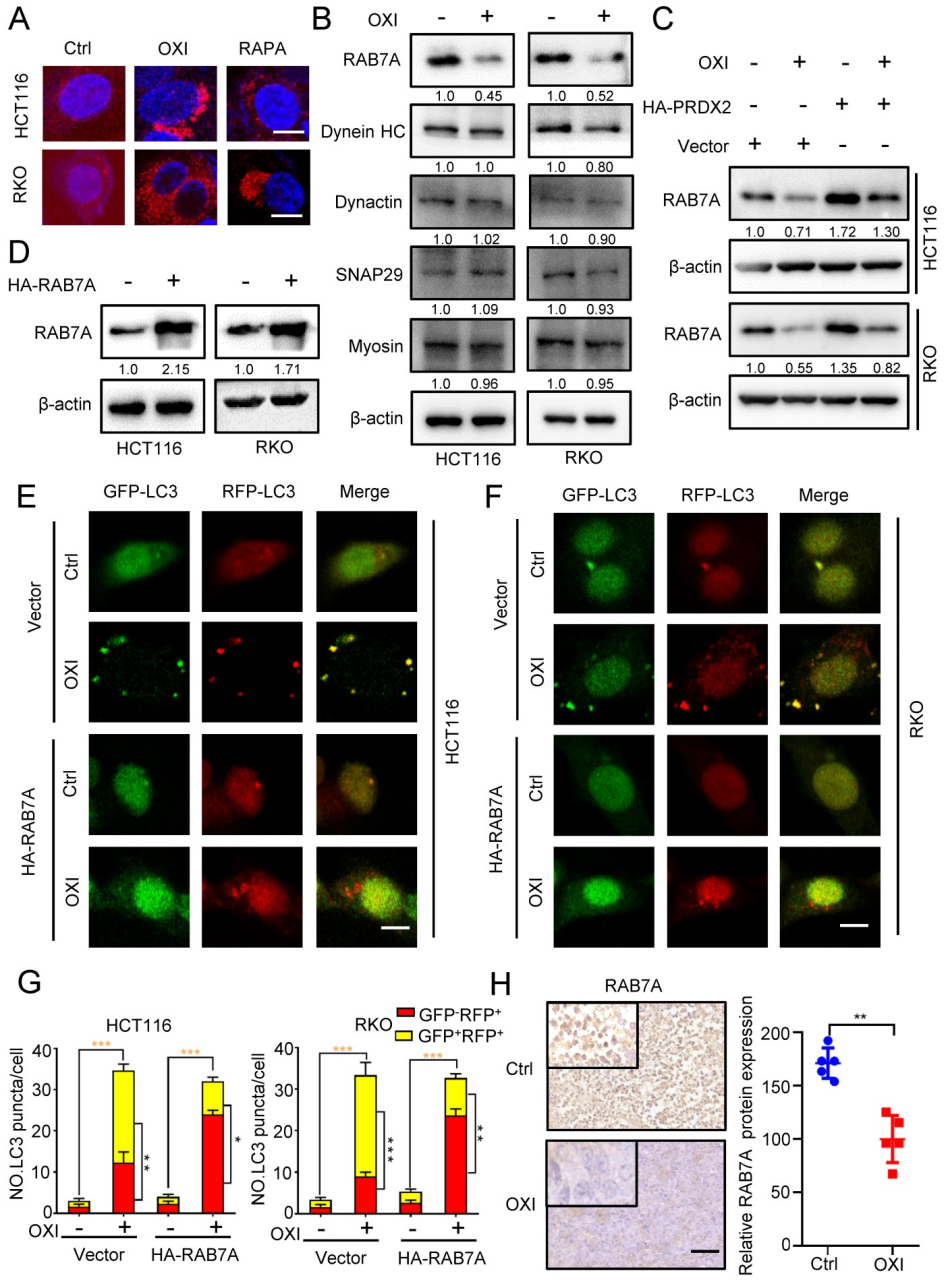
** OXI inhibits autophagosome-lysosome fusion by repressing PRDX2/RAB7A axis in CRC cells.** (A) RKO and HCT116 cells were treated with OXI (30 μM) or RAPA (100 nM) for 24 h, and then dyed with LysoTracker (75 nM) for 30 min. (B) Western blotting analysis of Myosin, SNAP29, Dynactin, Dynein HC and RAB7A treated with or without OXI (30 μM). (C) Western blotting analysis of RAB7A in HCT116 and RKO cells transfected with Vector or HA-PRDX2 and treated with OXI (30 μM). (D) Western blotting analysis of RAB7A protein level in HCT116 and RKO cells transfected with HA-RAB7A plasmids for 48 h. (E-F) HCT116 and RKO cells were transiently transfected with mRFP-GFP-LC3 and HA-RAB7A, then treated with OXI (30 μM). Scale bars, 10 μm. (G) Quantification of the ratio of yellow puncta (autophagosome) versus red puncta (autolysosome). (H) The expression level of RAB7A in xenografts was evaluated by IHC. Scale bar: 50 μm. All data are means ± SD.

As depicted in Fig. 6A, the lysosomal content revealed an obvious increase upon OXI treatment, suggesting that lysosomal mass might not be responsible for OXI-mediated inhibition of lysosome-autophagosome fusion. Immunoblotting assays were then used to examine the expression of proteins associated with lysosome-autophagosome fusion, including Myosin, SNAP29, Dynactin, Dynein HC and RAB7A 21, 22. Intriguingly, the expression of RAB7A was markedly decreased upon OXI treatment, whereas other proteins exhibited no obvious alterations (Fig. 6B). To further elucidate the role of PRDX2 in modulating RAB7A protein level, we performed immunoblotting analysis and found that the protein level of PRDX2 and RAB7A was positively correlated (Fig. 6C). We next asked whether the downregulation of RAB7A is involved in OXI-inhibited autolysosome formation. Using the tandem monomeric mRFP-GFP tagged LC3, the decreased GFP^+^RFP^+^ signal and increased GFP^-^RFP^+^ signal were observed in CRC cells overexpressing RAB7A under OXI treatment (Fig. 6D-G), which demonstrated that OXI inhibits the fusion of lysosome and autophagosome through downregulating RAB7A. Indeed, OXI-treated xenografts exhibited weaker RAB7A staining (Fig. 6H). Collectively, our findings demonstrate that downregulation of RAB7A mediated by PRDX2 decrease is responsible for OXI-induced autophagy arrest.

In addition, we further evaluated whether OXI could sensitize CRC cells to oxaliplatin, one of the first-line chemotherapy drugs for CRC treatment 23. As indicated, OXI plus oxaliplatin significantly repressed both growth (Fig. S5A) and proliferation (Fig. S5B and S5C) of CRC cells compared with oxaliplatin treatment alone, indicating that OXI can act synergistically with oxaliplatin to suppress CRC cell growth.

## Discussion

**Figure 7:**
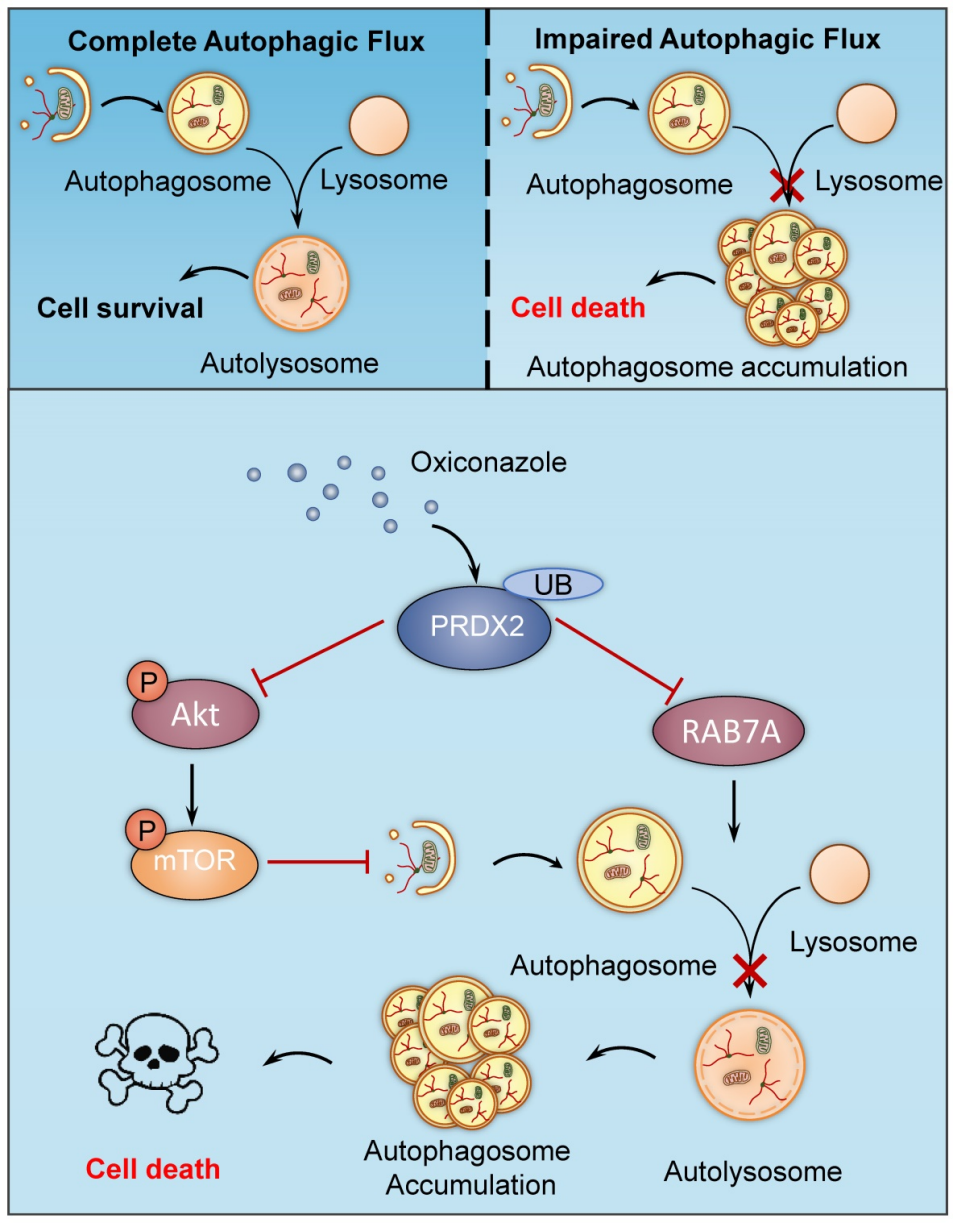
** A proposed model of the anti-CRC effects of Oxiconazole.** OXI triggers the downregulation of PRDX2 to initiate autophagy by inactivating the Akt/mTOR pathway and inhibit RAB7A-mediated fusion of autophagosome and lysosome, which results in accumulation of autophagosomes and subsequent growth suppression of CRC cells.

In this study, we have demonstrated the mechanisms involved in OXI-induced autophagy and apoptosis for suppressing growth of CRC cells. We found that OXI promotes autophagy initiation by inhibiting the Akt/mTOR axis mediated by PRDX2 downregulation, which simultaneously contributes to the disruption of RAB7A-mediated autophagosome-lysosome fusion, leading to subsequent autophagy arrest (Fig. 7). Indeed, OXI-mediated autophagy arrest is required for its tumor suppressive effect on CRC cells. These findings propose a novel mechanism for OXI-mediated CRC suppression with a focus on the role of PRDX2 in modulating autophagy.

The role of autophagy in tumor development and tumor therapy response varies widely and is likely context-dependent 24. Autophagy constitutes a stress adaptation process that protects cells from death under certain circumstances, whereas in other conditions it serves as an alternative cell-death pathway 25. Here, we initially demonstrated ROS-mediated apoptosis and induction of autophagy in OXI-treated CRC cells. Autophagy contributes to cell death in an apoptosis-dependent or -independent manner according to previous study 26. In line with our findings, Ziyuglycoside II induces cell death by triggering autophagy and apoptosis in CRC cells 27. However, in a glioblastoma model, Regorafenib stimulates lethal autophagy independent of apoptosis 8. There are also reports that autophagy exhibits protective role against tumor suppression, suggesting a strategy of combinatorial use with autophagy inhibitors to achieve a better therapeutic efficacy for cancer treatment 11, 28. These previous findings together with our present work suggest that autophagy may be involved in promoting survival or death of cancer cell in response to different conditions, which is likely to be model and drug-dependent.

Autophagy arrest attributes to the blocked autophagic flux, which leads to continuous accumulation of autophagosomes and may subsequently promote cell death 29. The Rab protein belongs to the Ras-like GTPase superfamily and regulates the autophagosome-lysosome fusion 30. Numerous Rab proteins have been shown to participate in various stages of autophagy 31. For instance, RAB11A was identified to regulate autophagy arrest in a previous study 8. Of note, RAB7A plays a key role in autophagosome maturation 32, 33, and overexpression of RAB7A promotes cancer progression 34-36. Consistently, our present study demonstrated that OXI impaired autophagic flux by RAB7A downregulation, and the blockage of autophagic flux could be partially reversed by enforced exogenous expression of RAB7A in OXI-treated CRC cells. Moreover, we found that the protein level of PRDX2 and RAB7A was positively correlated in CRC cells, but further in-depth studies are required to demonstrate the regulatory pattern.

PRDX2, an important member of the peroxiredoxin family which acts as enzymatic antioxidant proteins, has been found to be upregulated in several cancer types, including gastric, colorectal, and non-small cell lung cancer (NSCLC) 19, 37-41. Interestingly, overexpression of PRDX2 is correlated with tumorigenesis, tumor progression and drug resistance 42, 43. Previous studies have found that PRDX2 protein levels were higher in human CRC tissues and were closely correlated with CRC progression, suggesting the potential of targeting PRDX2 for the clinic treatment of CRC 43. In addition, a recent study demonstrated that a novel circular RNA DIDO1 could specifically bind to PRDX2 and promote its ubiquitination and degradation in a RBX1-dependent way, which inhibited its downstream signaling pathways to suppress tumor progression 44. In the present study, we demonstrated that OXI treatment reduced PRDX2 protein levels through proteasomal degradation, thus leading to autophagy arrest and subsequent growth suppression of CRC cells. In addition, restoring PRDX2 expression significantly decreased autophagy levels and relieved the suppressive role of OXI on CRC cell growth, indicating that OXI displays the anti-CRC effect partially by modulating PRDX2. Further studies regarding the detailed mechanisms of OXI-induced degradation of PRDX2 will hopefully prove the therapeutic potential of PRDX2 for CRC treatment.

The Akt/mTOR signaling is the main negative regulator of autophagy, and participates in regulating tumor proliferation 16, 45. In the present study, OXI restrained the Akt/mTOR axis, as demonstrated by the decreased phosphorylation levels of Akt and mTOR. In addition, our results showed that inactivation of Akt/mTOR in OXI-treated CRC cells was attributed to downregulation of PRDX2, whose expression was positively associated with Akt phosphorylation 18. These findings showed that OXI downregulated PRDX2 protein levels and inhibited the phosphorylation of Akt/mTOR, indicating that the PRDX2/Akt/mTOR axis is associated with OXI-induced autophagy, although the details need further investigation. Overall, our results indicate that targeting the PRDX2/Akt/mTOR axis is worthy of further exploration as a potential treatment option for CRC.

Taken together, our study demonstrates that autophagy arrest induced by PRDX2 inhibition may be the primary contributing factor for growth suppression in response to OXI treatment in CRC cells. These findings provide novel evidence into the mechanism of action of OXI, implying OXI as a potential repurposed drug for CRC treatment and highlighting the concept that autophagy may hold promise as a therapeutic target in cancer.

## Supplementary Material

Supplementary figures.
