# Supplementary material for: Repurposing Oxiconazole against Colorectal Cancer via PRDX2-mediated Autophagy Arrest
Source: Int J Biol Sci. 2022 May 21;18(9):3747–61. doi: 10.7150/ijbs.70679 (PMC9254464; doi:10.7150/ijbs.70679)

Figure S1. OXI displays no obvious side effects on mice. (A) H&E staining of the kidney, liver, lung, spleen and heart in mice treated with OXI (50 mg/kg/day) or vehicle. Scale bar: 50 μm.

Figure S2. OXI triggers autophagy in CRC cells. (A) Time course detection of LC3 levels in HCT116 and RKO cells treated with OXI (30 μM) for 6, 12, 18, 24 h by western blotting analysis. (B) Western blotting analysis of LC3 expression in CRC cells transfected with si*BECN1* or si*Scramble* and treated with OXI (30 μM). (C-E) Immunofluorescent analysis of endogenous LC3B puncta in HCT116 and RKO cells transfected with si*ATG5* and treated with OXI (30 μM), and quantification of LC3 puncta. Scale bars, 10 μm. (F) Co-IP analysis of the interaction between Bcl2 and Beclin1 in HCT116 and RKO cells treated with OXI (30 μM). All data are means ± SD.

Figure S3. OXI promotes the initiation of autophagy by inhibiting Akt/mTOR axis.

(A) The phosphorylated mTOR, Akt, 4EBP1 and p70S6K were detected by western blotting in cells treated with OXI for 24 h. (B) HCT116 and RKO cells were transiently transfected with CA-Akt and treated with OXI (30 μM). Western blotting was used to examine the protein levels of p-Akt and lipidated LC3. (C-D) HCT116 and RKO cells were treated as in B, and immunofluorescent analysis of LC3 puncta were performed. All data are means ± SD.

Figure S4. Downregulation of PRDX2 triggers autophagy in CRC cells. (A) Immunofluorescent analysis of LC3B puncta in HCT116 and RKO cells transfected with HA-PRDX2 plasmids and treated with OXI. Scale bars, 10 μm. (B) Endogenous LC3 puncta in RKO and HCT116 cells transfected with si*PRDX2* for 48 h. Scale bars, 10 μm. (C) The protein levels of PRDX2 and LC3 in HCT116 and RKO cells transfected with si*PRDX2* for 48 h. All data are means ± SD.

Figure S5. OXI enhances the anti-CRC efficacy of oxaliplatin. (A) Cell viability of HCT116 and RKO cells treated with the different concentrations of OXI and oxaliplatin for 24 h. (B-C) The colony formation assay of HCT116 and RKO cells treated with OXI (30 μM) in combination with oxaliplatin (10 μM). All data are means ± SD.

Figure S1. OXI displays no obvious side effects on mice.

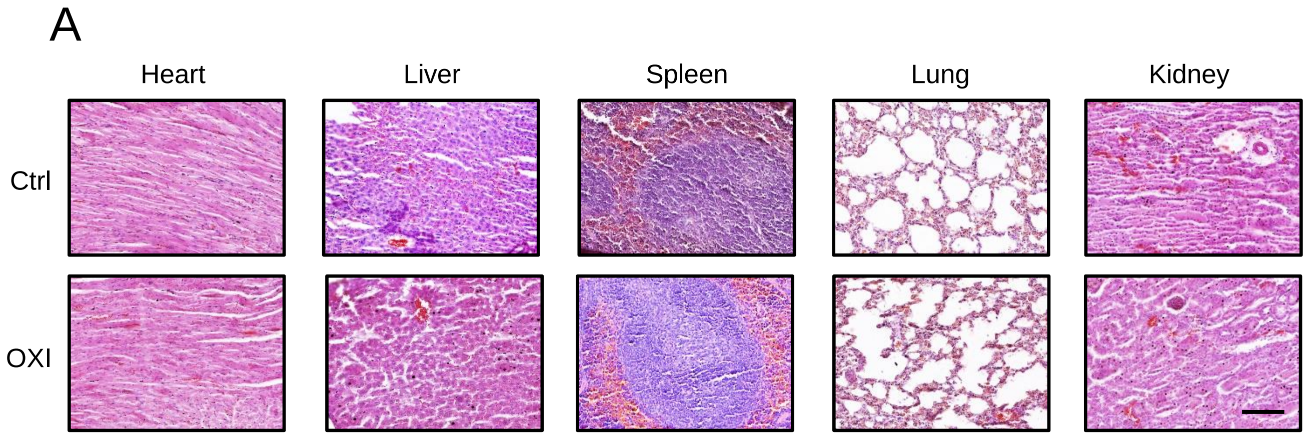

Figure S2. OXI triggers autophagy in CRC cells.

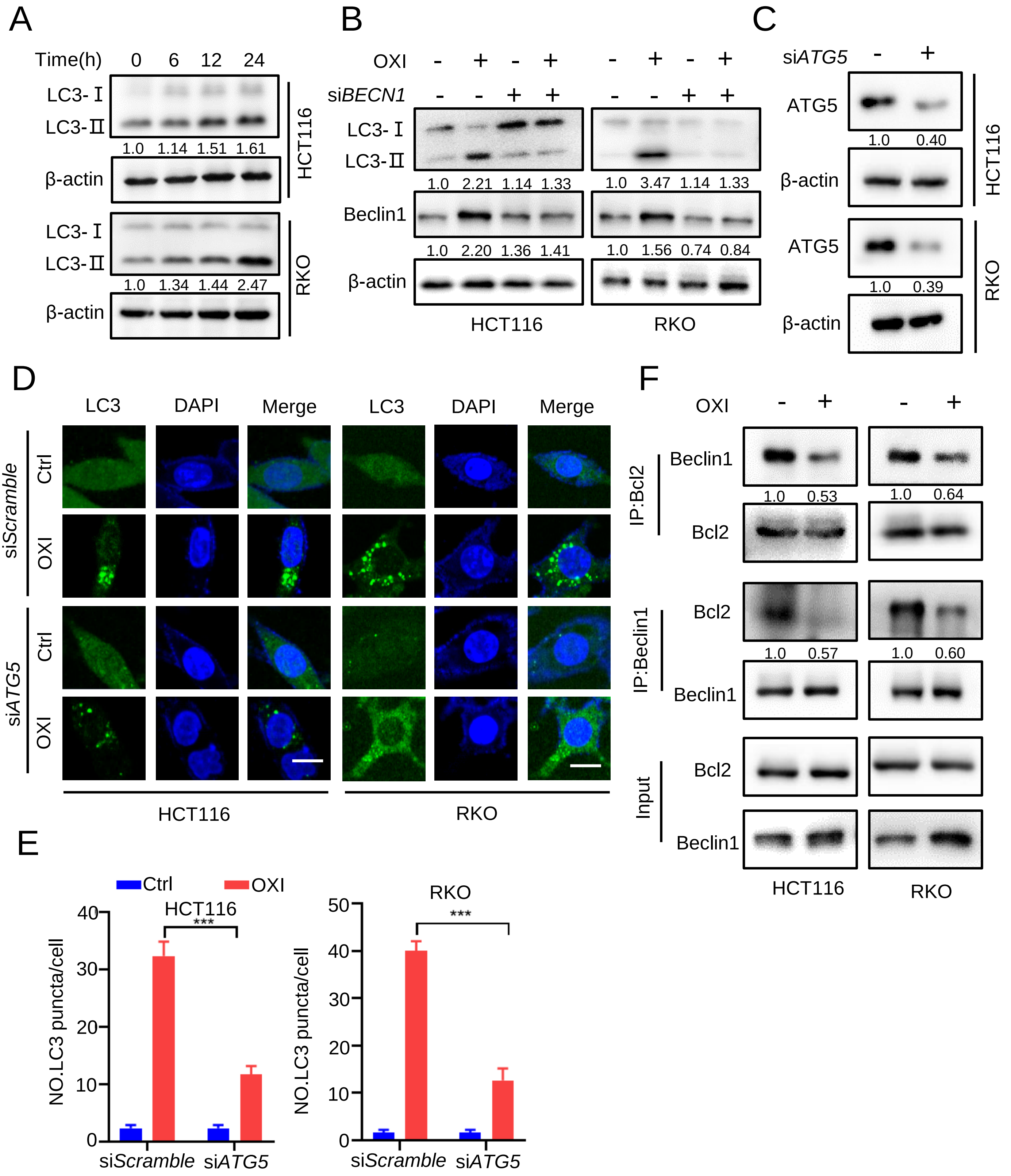

Figure S3. OXI promotes the initiation of autophagy by inhibiting Akt/mTOR axis.

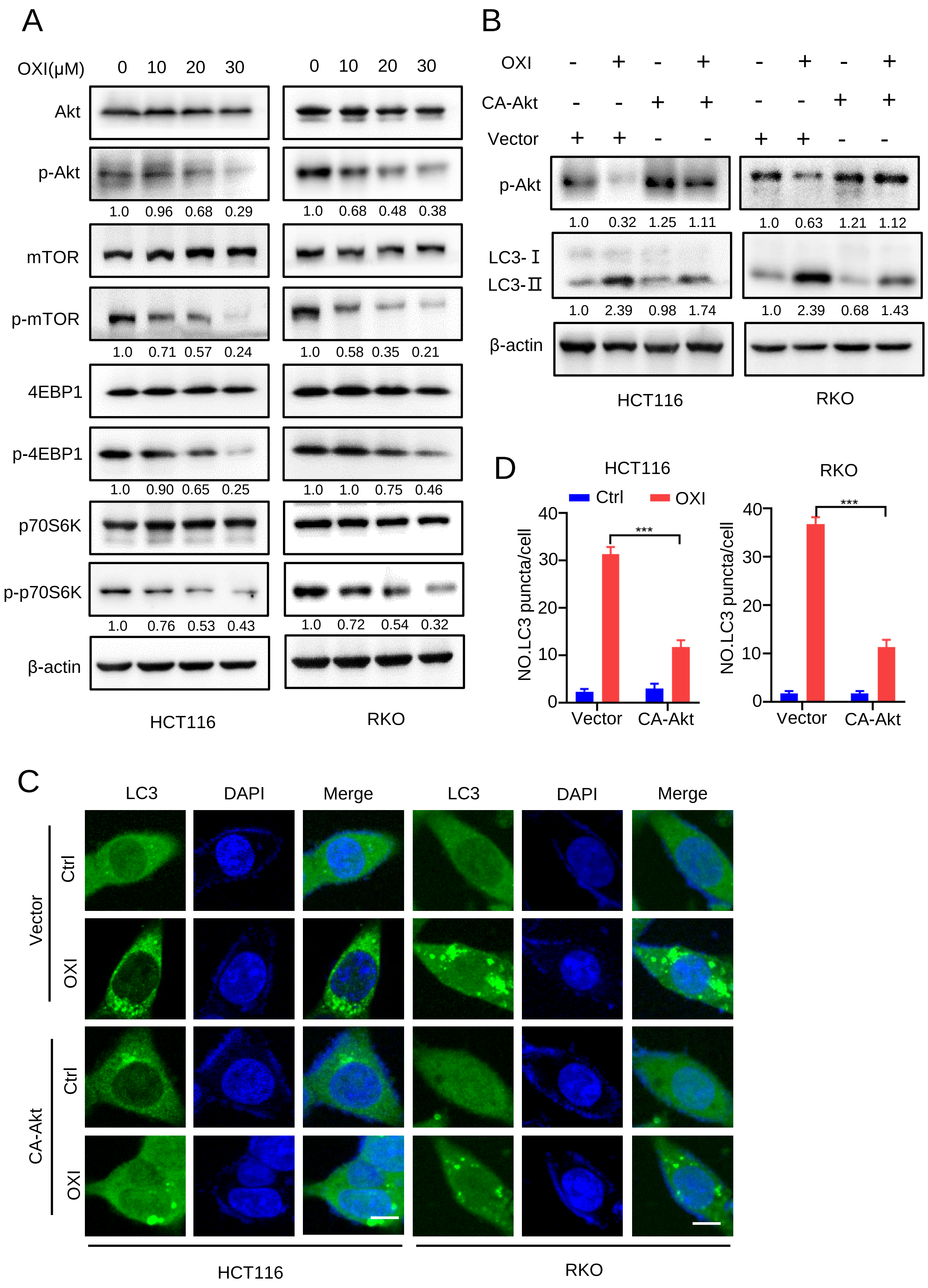

Figure S4. Downregulation of PRDX2 triggers autophagy in CRC cells.

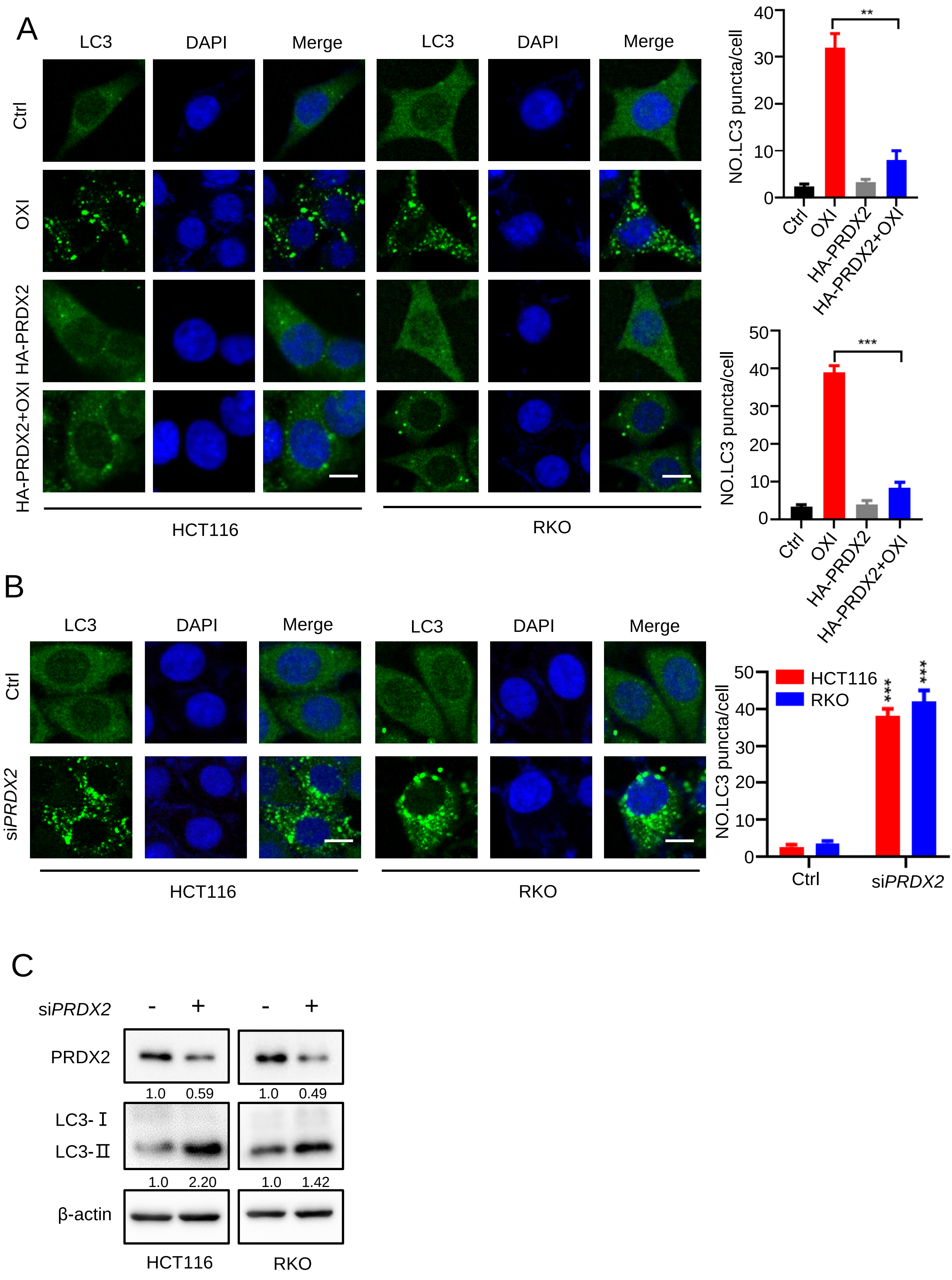

Figure S5. OXI enhances the anti-CRC efficacy of oxaliplatin.

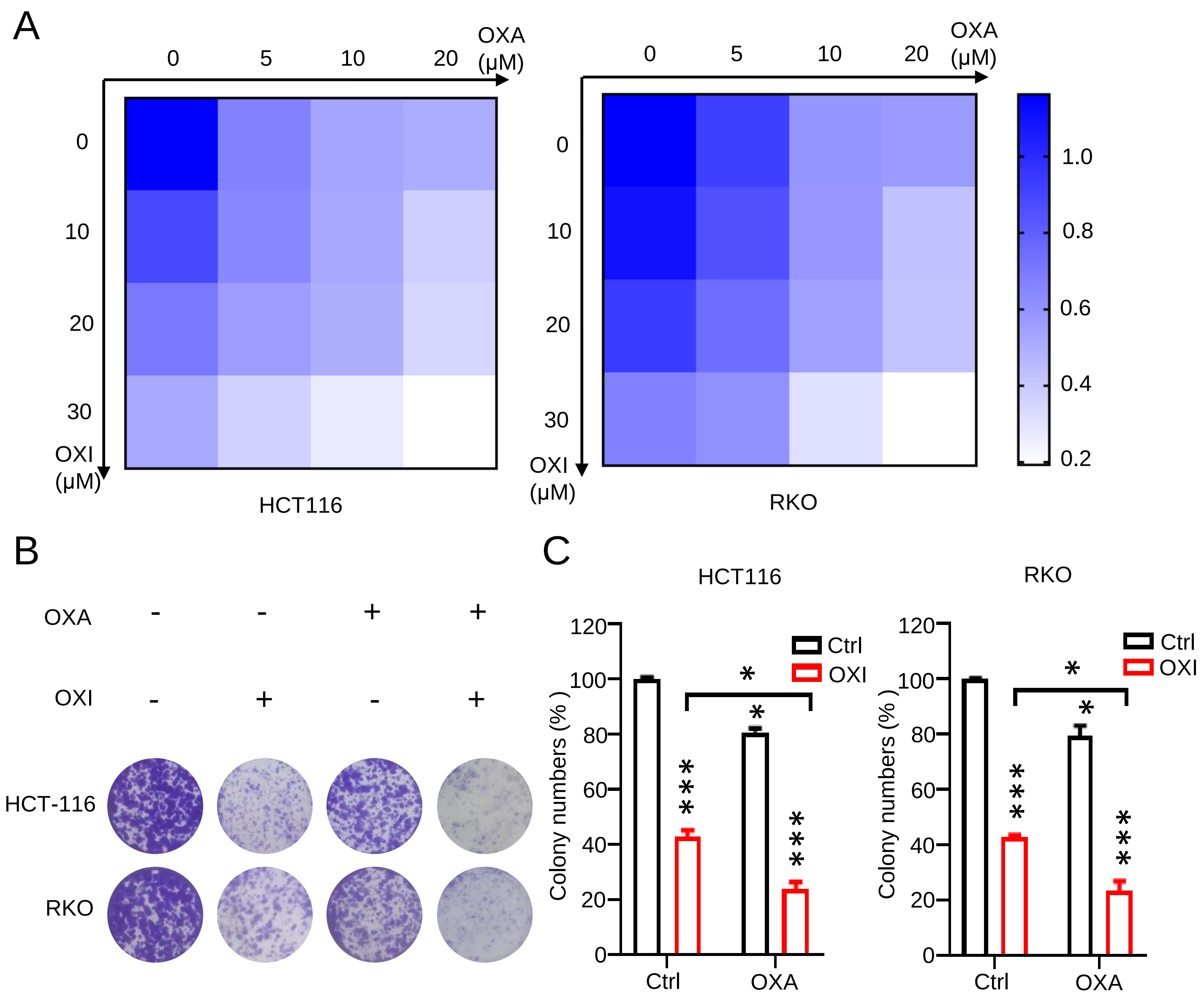

Supplement: Supplementary file 1 — Supplementary figures. [file ijbsv18p3747s1.pdf]
